# Tungsten Disulfide Nanotube-Modified Conductive Paper-Based Chemiresistive Sensor for the Application in Volatile Organic Compounds’ Detection

**DOI:** 10.3390/s21186121

**Published:** 2021-09-12

**Authors:** Song-Jeng Huang, Philip Nathaniel Immanuel, Yi-Kuang Yen, Ching-Lung Yen, Chi-En Tseng, Guan-Ting Lin, Che-Kuan Lin, Zhong-Xuan Huang

**Affiliations:** 1 Department of Mechanical Engineering, National Taiwan University of Science and Technology, Taipei 106, Taiwan; sgjghuang@mail.ntust.edu.tw (S.-J.H.); D10603816@mail.ntust.edu.tw (P.N.I.); 2 Department of Mechanical Engineering, National Taipei University of Technology, Taipei 106, Taiwan; t106300310@ntut.org.tw (C.-L.Y.); t106300308@ntut.org.tw (C.-E.T.); t106300324@ntut.org.tw (G.-T.L.); t106300317@ntut.org.tw (C.-K.L.); t106300304@ntut.org.tw (Z.-X.H.)

**Keywords:** graphene, INT-WS_2_, response time, chemiresistor, VOC gas, limit of detection

## Abstract

Toxic and nontoxic volatile organic compound (VOC) gases are emitted into the atmosphere from certain solids and liquids as a consequence of wastage and some common daily activities. Inhalation of toxic VOCs has an adverse effect on human health, so it is necessary to monitor their concentration in the atmosphere. In this work, we report on the fabrication of inorganic nanotube (INT)-tungsten disulfide, paper-based graphene–PEDOT:PSS sheet and WS_2_ nanotube-modified conductive paper-based chemiresistors for VOC gas sensing. The WS_2_ nanotubes were fabricated by a two-step reaction, that is oxide reduction and sulfurization, carried out at 900 °C. The synthesized nanotubes were characterized by FE-SEM, EDS, XRD, Raman spectroscopy, and TEM. The synthesized nanotubes were 206–267 nm in diameter. The FE-SEM results show the length of the nanotubes to be 4.5–8 µm. The graphene–PEDOT:PSS hybrid conductive paper sheet was fabricated by a continuous coating process. Then, WS_2_ nanotubes were drop-cast onto conductive paper for fabrication of the chemiresistors. The feasibility and sensitivity of the WS_2_ nanotube-modified paper-based chemiresistor were tested in four VOC gases at different concentrations at room temperature (RT). Experimental results show the proposed sensor to be more sensitive to butanol gas when the concentration ranges from 50 to 1000 ppm. The limit of detection (LOD) of this chemiresistor for butanol gas was 44.92 ppm. The WS_2_ nanotube-modified paper-based chemiresistor exhibits good potential as a VOC sensor with the advantages of flexibility, easy fabrication, and low fabrication cost.

## 1. Introduction

The WS_2_ nanotubes form a transition metal dichalcogenide (TMD) material with S–W–S layers bonded by weak van der Waals forces. In a nanotube, every W atom is bonded with three S atoms in a hexagonal shape. WS_2_ has an indirect bandgap of 1.6 eV [1,2]. 2D materials such as graphene transition materials have a tunable bandgap, where the number of layers reduces the indirect bandgap transit to direct bandgap [3]. Theoretical predictions show that the absorbed molecules on the monolayer or few layers on WS_2_ could change the bandgap, which causes the changes in electrical and optical properties [4]. This advantage makes WS_2_ attractive in various fields, such as photo-detection, chemical sensing, and flexible electronics [5,6]. The properties of WS_2_ combined with graphene are the most studied in the field of gas sensing [4].

Any molecule that has carbon and hydrogen in its structure is categorized as an organic compound. Organic compounds are classified into many types, and any organic compound which is in the form of gas or liquid under normal temperature and pressure is referred to as a volatile organic compound (VOC) [7]. In total, there are 50–300 different kinds of VOCs. These VOCs are released from everyday materials such as plastics, paints, wood, and food, and activities such as cutting grass and combustion [8]. Not all VOCs are toxic, but inhaling these gases can cause problems to human health [7]. Studying the exhaled concentration of VOC can help with the detection of disease. These exhaled VOC concentrations can be utilized as a biomarker to detect illnesses such as kidney malfunctioning, lung cancer, and asthma [9,10]. Methodologies can be developed for real-time monitoring to help with early disease detection [11]. In addition, given that these gases are commonly found in our environment but are unsafe to inhale, it is important to monitor the concentration of VOCs in our living environment. Techniques such as gas chromatography and mass spectroscopy can detect concentrations as low as subparts per million [9,11], but they require huge expensive and complicated detectors. A compact device with high sensing efficiency, low cost, simple design, and easily available is needed to overcome this issue and fabricate an effective VOC sensor [12]. A compact VOC sensing device would ideally be a small component, such as a resistor, capacitor, or a transistor that can sense gas molecules at ambient temperatures and provide the potential or frequency [13]. On the other hand, e-noise-based sensors have been reported for sensing [14]. Sensors such as resistors have varied conductivities under different gas environments [15], and this field is open to the introduction of new techniques and materials for the fabrication of stable, highly sensitive sensors with fast response and recovery times. Thus, materials, such as nanomaterials, combined with polymers are recommended for their high surface-to-volume ratio [16].

Materials which have different physical properties under different gas environments can be used for effective sensing [8]. Nanomaterials, such as 2D graphene, are quite effective for detecting low concentrations [16,17]. Materials such as perylene diimides have also been used for sensing some toxic gases, such as hydrazine and ammonia [18]. Graphene has excellent electrical, thermal, and mechanical properties, as well as a zero bandgap, which makes it a good choice for sensing. It also has high electron mobility (2.5 × 10^5^ cm^2^ V^−1^ S^−1^) at RT, leading to low power consumption, low electrical noise, high surface-to-volume ratio, tailorable chemical functionalization, and high open porosity [9,11]. Graphene can detect even a single molecule [8].

Thus, graphene plays an important role in the detection of very low concentrations of VOC. Graphene has demonstrated a highly sensitive response to NH_3_ and NO types of gases. However, the response time is the drawback of this material, and the fabrication of sensors based on nanomaterials is a difficult process [19]. Reports suggest that the response time and recovery time can be improved by combining graphene with a polymer [15].

Some polymers, such as PEDOT, are suitable for sensing gases and vapor. Combining PEDOT with graphene enhances the sensitivity, signal-to-noise ratio, and selectivity, and reduces the response time [8]. Studies have shown that a combined graphene and polymer sensor for VOC sensing has enhanced sensitivity [20,21] and rapid response and recovery times [21,22]. The PEDOT:PSS is an electrically conductive polymer that has a low redox potential and good environmental stability. Many researchers have used PEDOT:PSS for the fabrication of carbon nanotubes and graphene, to obtain fast response times [19]. The combination of graphene quantum dots with PEDOT:PSS can detect low concentrations (1–100 ppm) at RT. The combination of graphene, PEDOT:PSS, and TMD is the best choice for our gas sensor.

This study aims to fabricate a WS_2_ NT-modified conductive paper-based chemiresistor for VOC gas sensing at RT. The response of this sensor to four different VOCs, namely, acetone, methanol, toluene, and n-butanol, is studied under different concentrations.

## 2. Materials and Methods

### 2.1. Materials

The WO_3_ powder (diameter of < 100 nm) was obtained from Sigma Aldrich (Steinheim, Germany) while the Acetone [((CH_3_)_2_CO)], methyl alcohol [(CH_3_OH)], toluene [(C_6_H_5_CH_3_)], and n-butanol [(C_4_H_9_OH)] were obtained from Nihon Shiyaku Industries (Taipei, Taiwan).

### 2.2. Preparation of the WS_2_ Nanotube

The WS_2_ nanotubes were synthesized by solid–gas reaction from WO_3_ powder with the dimension of <100 nm. The WO_3_ powder was dispersed uniformly and kept under a 1%H_2_/99%N_2_ gas atmosphere for 1 h at 900 °C using a flow rate of 400 mL min^−1^. This H_2_ gas reduced the WO_3_ into the intermediate phase of WO_3−x_, as shown in Equation (1). After this reduction process, 1%H_2_/99%N_2_ and H_2_S gas was introduced into the reaction chamber for 1 h to sulfurize the reduced WO_3−x_. This sulfurization reaction produced WS_2_ [23,24]. The sulfurization reaction process is shown below. WS_2_ nanotubes were taken out from the reaction chamber after it cooled down to RT.

WO_3_ (s) + H_2_ (g) → WO_3−x_ + xH_2_O + (1 − X) H_2_; (1)

WO_(3−x)_ + (1 − x) H_2_ + H_2_S → WS_2_ + (3 − x) H_2_O. (2)

### 2.3. Preparation of the Graphene Sheet

**Scheme 1:**
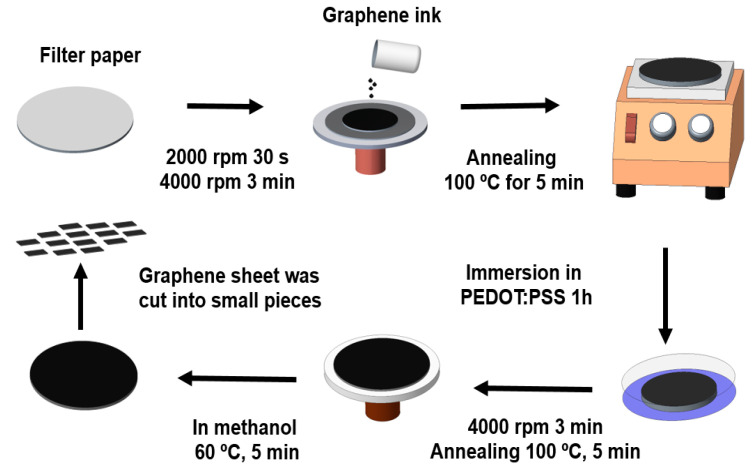
Fabrication and modification of the graphene–PEDOT:PSS.

The graphene–PEDOT:PSS-modified paper was fabricated as reported in [25]. The Whatman No. 3 filter paper was initially washed with three drops of Tween mixed with deionized water. After this washing process, the paper was immersed and sonicated in ethanol for 15 min. The paper was then washed with deionized water and dried in an oven at 60 °C. The graphene ink was coated uniformly on the filter paper by using a spin-coating apparatus at 4000 rpm for 3 min. The graphene-coated paper was annealed at 100 °C for 5 min. After annealing, the graphene paper was immersed in the PEDOT:PSS solution for 60 min. For uniform distribution, the sheet was spin-coated for another 3 min at 4000 rpm and annealed at 100 °C for 5 min. The graphene–PEDOT:PSS-modified paper was treated with methanol at 100 °C for 5 min and air-dried for enhanced conductivity. The fabrication steps for graphene–PEDOT:PSS are shown in Scheme 1.

### 2.4. Preparation of the Gas Sensing Device

**Scheme 2:**
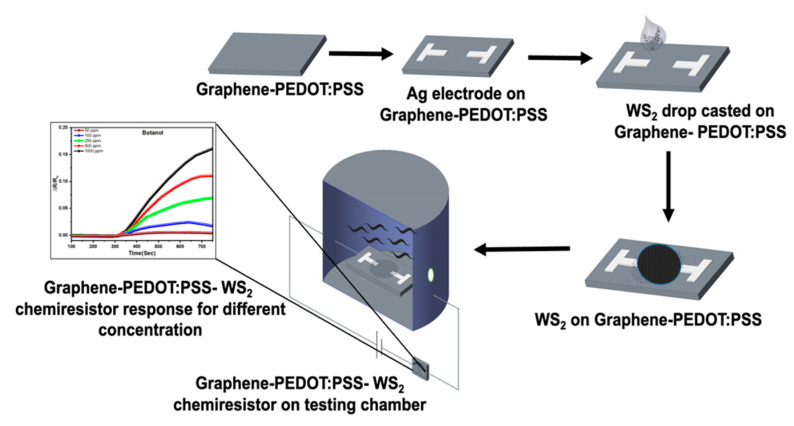
Fabrication and testing of the graphene–PEDOT:PSS/WS_2_ resistor.

The VOC chemiresistor was fabricated by a simple technique of drop-casting of as-synthesized WS_2_ nanotubes on the graphene–PEDOT:PSS conductive sheet. The graphene–PEDOT:PSS conductive sheet was then cut into small sheets with a length and width of 2 and 1 cm, respectively. Ag paste was used as the electrode, as shown in the schematic diagram. Synthesized WS_2_ nanotubes (0.1 g) were dispersed in 5 mL of ethanol by sonication. The dispersed WS_2_ nanotubes were drop-casted between the electrodes to form the chemiresistor setup. After the drop casting, the device was placed on a hot plate for 10 min at 70 °C to allow complete evaporation of the ethanol. The resultant paper-based graphene–PEDOT:PSS/WS_2_ nanotube chemiresistor was kept inside the gas sensing chamber. All steps are shown in Scheme 2. The completely fabricated paper-based graphene–PEDOT:PSS/WS_2_ nanotube chemiresistor’s images are presented in Figure S1 (Supplementary Materials).

### 2.5. Test Condition

The fabricated paper-based graphene–PEDOT:PSS/WS_2_ chemiresistor was kept inside the testing chamber, which was connected to a measuring device (Keithley 2400) under a normal dry air environment for the baseline calculation for 5 min. The response was monitored using a Keithley 2400 multichannel source meter. After 5 min, the VOCs were allowed into the chamber. Experiments were carried out using different concentrations (50, 100, 250, 500, and 1000 ppm) to study the response and the sensitivity at RT with 70% relative humidity. Inorganic semiconductor-based sensors exhibit long lifespan, stability, and reliability at high humidity and RT [26,27]. The same five concentrations were studied for the four VOCs (i.e., acetone, methanol, toluene, and n-butanol). The concentration inside the chamber was calculated using Equation (3):

(3) $$ppm=\frac{VOC\times \rho (VOC)}{MW(VOC)}\frac{R\times T}{P\times V}$$

where *ρ* is the density, *MW* is the molecular weight of the VOC, *R* is the gas constant, *T* is the temperature (26 °C), *P* is the pressure, and *V* is the volume of the chamber [28]. In this study, the responses to all VOCs for three cycles were evaluated. Different resistance values were obtained for different materials as a response to different gases. Equation (4) is used to convert the variation in the resistance response into amplitude and generalize the response of the sensor, where *R_g_* is the resistance in the VOC environment, and *R_b_* is the resistance in the dry air stream. The gas sensing study was performed at RT.

(4) $$Amplitude=\frac{R_{g}-R_{b}}{R_{b}}$$

## 3. Results and Discussion

### 3.1. WS_2_ Nanotube Characterization

**Figure 1:**
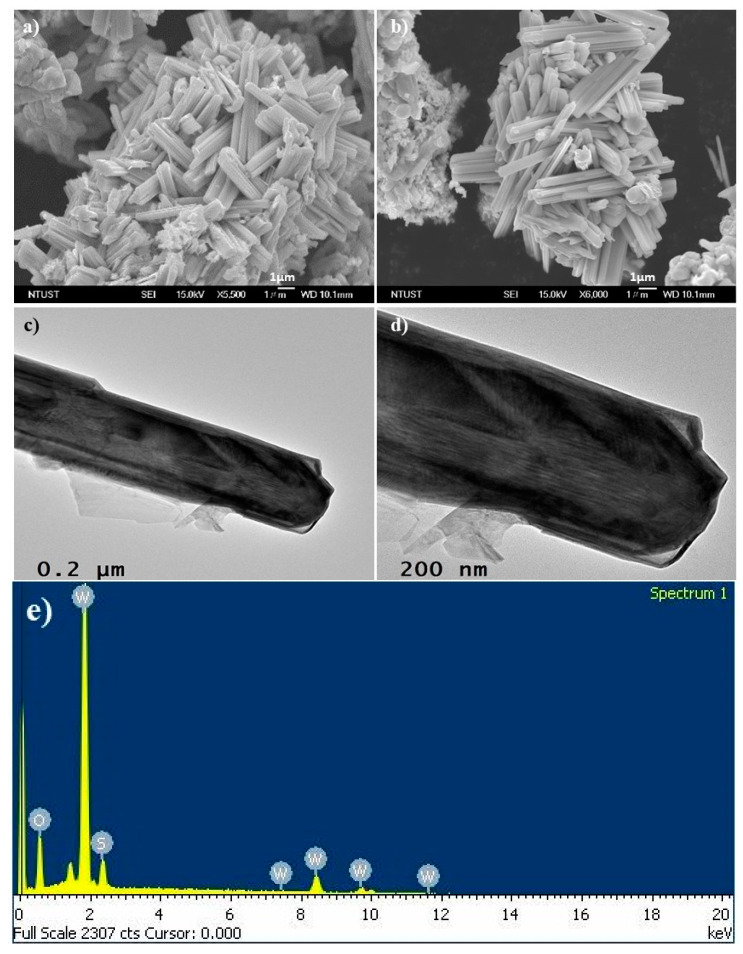
(**a**,**b**) FE-SEM images for the WS_2_ nanotubes, (**c**,**d**) TEM images of the synthesized WS_2_ nanotubes, and (**e**) EDS results for WS_2_ nanotubes.

The synthesized WS_2_ nanotubes were analyzed using FE-SEM, EDS, XRD, Raman analysis, and TEM. The FE-SEM, TEM, and EDS results are presented in Figure 1. Figure 1a,b shows the surface morphology of the as-synthesized WS_2_ nanotubes. The structure shows that the synthesized nanotubes bind together and form bundles. The nanotubes’ orientation is very random, and the surfaces are quite uniform and clear throughout the sample. The nanotubes were from 206.9 to 318 nm in diameter and 4.5–8.03 µm in length. The TEM images (Figure 1c,d) show that the synthesized individual WS_2_ nanotubes are about 318 nm in diameter, which agrees with the FE-SEM results. Figure S2 (Supplementary Materials) shows that the as-synthesized nanotubes are multiwall nanotubes.

**Figure 2:**
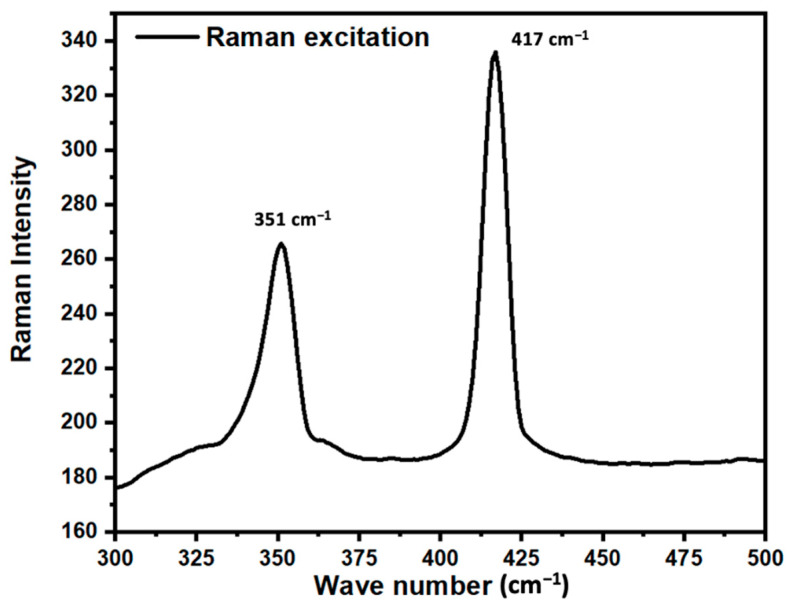
Raman excitation peaks of the WS_2_ nanotubes.

The EDS results (Figure 1e) confirm the presence of W and S in the nanotubes, and the presence of O was also detected. XRD results presented in Figure S3a (Supplementary Materials) show the comparative results between WO_3_ and as-synthesized WS_2_. The XRD results match with JCPDS PDF 84–1398 for WS_2_. The strong peak detected at 14.364° confirms the presence of WS_2_, and other peaks represent the intermediate phases of W_18_O_49_. The sharp and high-intensity peak at 14.36° confirms the high percentage of WS_2_ formed from WO_3_. The quantitative analysis was performed with the XRD results, and the results are shown in Supplementary Figure S3b. From the quantitative analysis, the percentages of O, S, and W are 9.7%, 12.8%, and 77.5% respectively, which also agrees with the EDS results. Figure 2 shows the Raman spectrum of the WS_2_ nanotubes at excitation wavenumbers of 351 and 417 cm^−1^, confirming the formation of WS_2_ in this synthesis process. The excitation peak at 351 cm^−1^ represents the longitudinal acoustic phonons LA(M) mode, which is a second-order Raman peak, and peak at 417 cm^−1^ represents the A_1g_(Γ) mode, the first-order Raman peak [3].

### 3.2. Gas Sensor Characteristics

**Figure 3:**
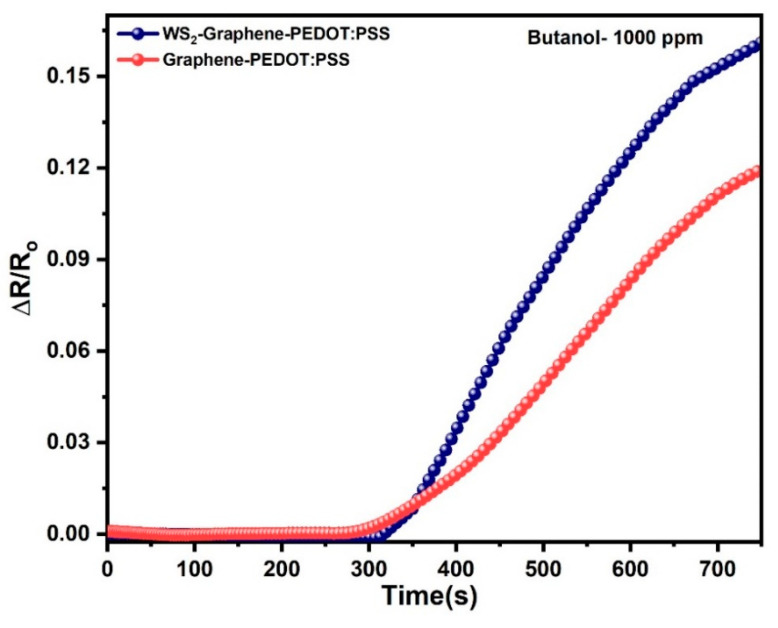
Response curve for butanol gas at the 1000 ppm concentration before and after the deposition of WS_2_ nanotubes.

Initially, we studied the response of the paper-based graphene–PEDOT:PSS sheet alone for butanol gas at a concentration of 1000 ppm at RT. Then, the paper-based graphene–PEDOT:PSS/WS_2_ chemiresistor was tested using the same concentration at RT. As can be seen in Figure 3, the response of the chemiresistor was 28% higher than that of the paper-based graphene–PEDOT:PSS sheet, showing that the inorganic WS_2_ nanotubes enhanced the sensitivity to VOCs. Initially the chemiresistor’s response was studied at open-air condition for 5 min. After 5 min of baseline correction, the VOC gas butanol was tested for 5 min at RT. The response time of the paper-based graphene–PEDOT:PSS/WS_2_ chemiresistor was 10 s shorter than that of the paper-based graphene–PEDOT:PSS sheet. Since filter paper is the base to fabricate the VOC sensor, the sensor is flexible in nature as filter paper.

**Figure 4:**
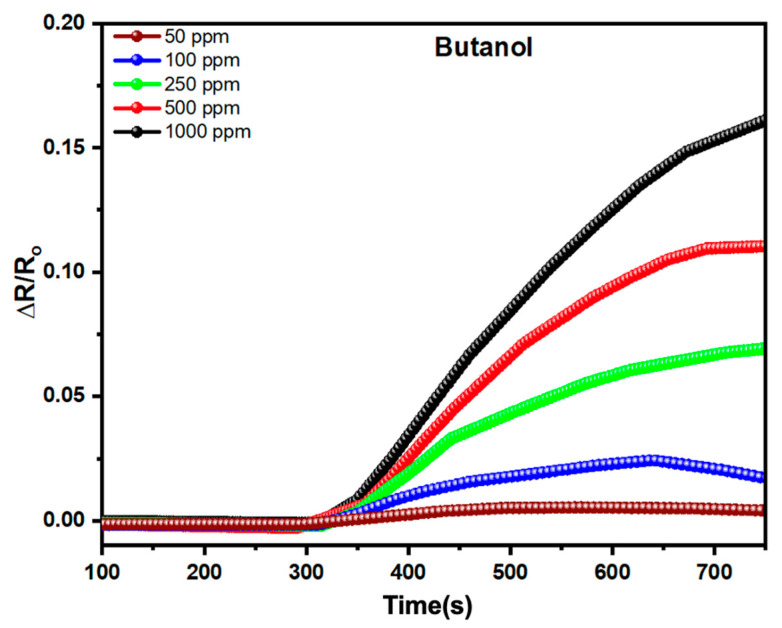
Paper-based graphene–PEDOT:PSS/WS_2_ nanotube chemiresistor response for n-butanol gas under different concentrations at RT.

The paper-based graphene–PEDOT:PSS/WS_2_ nanotube chemiresistor’s resistance variations and the response to the VOCs, namely, methanol, toluene, and butanol, were recorded. The chemiresistor’s response was studied for all four gases to study the gas sensor’s feasibility at RT. Figure 4 shows the response curve for butanol gas at different concentrations. The results showing the response to methanol and toluene gases are presented in Figure S4b,d (Supplementary Materials). From the results, it can be seen that the gas sensor showed a clearer and well-defined response to butanol gas than to the other VOCs tested in this experiment. The responses to acetone gas were not well-defined. The response to acetone gas was also lower compared with the response to toluene and the other VOCs. The responses of the paper-based graphene–PEDOT:PSS/WS_2_ nanotube chemiresistor were as follows: R _acetone_ < R _methanol_ < R _toluene_ < R _butanol_. The response to the toluene gas was 108.94% stronger than the response to the methanol gas at concentrations of 1000 ppm.

From the results presented in Figure 4 and Figure S4 (Supplementary Materials), it can be seen that the response was stronger when the concentration of the VOCs was high. When the concentration was low, the amplitude of the response was low. The response to 1000 ppm butanol gas was 48% stronger than the response to toluene gas. The sensitivity of the chemiresistor to butanol gas at low gas concentration (50 ppm) is stronger than for the other VOCs studied in this work. The response time for each VOC differed, and the recovery time for the different gases also changed. The response signal did not reach the saturation point for any of the VOCs tested, even at low concentrations.

The response of the WS_2_ nanotube-modified paper-based chemiresistor to acetone gas was comparatively weaker than for the other gases, and the response to different acetone concentrations was random. Responses were defined when methanol, toluene, and butanol gases were tested. The response time to 1000 ppm methanol was about 91 s. The response time at low concentrations was shorter than at high concentrations. The response times at 100 and 50 ppm were about 41 and 28.6 s, respectively. For methanol gas, the sensor demonstrated good reproducibility at all concentrations. The amplitude of the response signal remained the same regardless of the concentration and the number of cycles. The cyclic gas sensing results are shown in Figure S4b (Supplementary Materials). The response times at different concentrations are presented in Table S1 (Supplementary Materials). The response time was defined as the time to reach 90% [29] of the maximum total resistance changes of the paper-based graphene–PEDOT:PSS/WS_2_ nanotube gas sensors. Since the response for acetone was very random for all three cyclic studies and for all concentrations, the WS_2_ nanotube-modified paper-based chemiresistor is not suitable to detect acetone.

The WS_2_ nanotube-modified paper-based chemiresistor showed well-defied signals, and the response was strong at high gas concentrations and weaker at low gas concentrations. The response time varied with respect to the VOCs and their concentration. The toluene gas response was 109.25% higher than the methanol gas response at the 1000 ppm concentration. When the concentration was decreased to 50 ppm, the difference was 64% higher than the methanol gas response. The three-cycle response to toluene gas at five different concentrations is presented in Figure S4d (Supplementary Materials).

**Figure 5:**
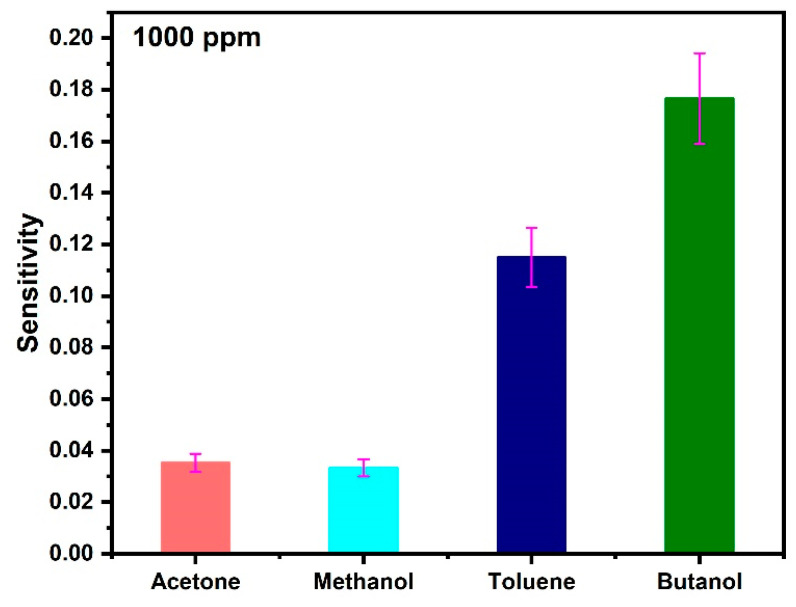
Histograms showing the acetone, methanol, toluene, and butanol gas responses at concentrations of 1000 ppm.

Finally, the response to n-butanol was studied using the same set of concentrations for three cycles. The results are presented in Figure S4c (Supplementary Materials). At low ppm (50 ppm), the chemiresistor exhibited a stronger response to n-butanol than to methanol, toluene, or acetone. The response was 90.6% higher than the methanol gas response at 50 ppm. The response signals were also more well-defined than the other response signals. The amplitude of the response signal was enhanced by about 1.5-fold over the toluene gas response at 1000 ppm. Figure 5 shows a comparison of the responses at 1000 ppm for acetone, methanol, toluene, and n-butanol. Figure S5 (Supplementary Materials) shows a comparison for all the different concentrations for the four VOC gases. Unlike other responses, the resistor’s response dropped down to baseline much faster when the VOC gases were stopped. When the chemiresistor was exposed to other gases, the recovery time was longer. The response to butanol gas for all three cycles was pretty much the same in the three cyclic studies. There was only a slight variation in the response amplitude for the butanol gas, which shows that the chemiresistor had good reproducibility.

**Figure 6:**
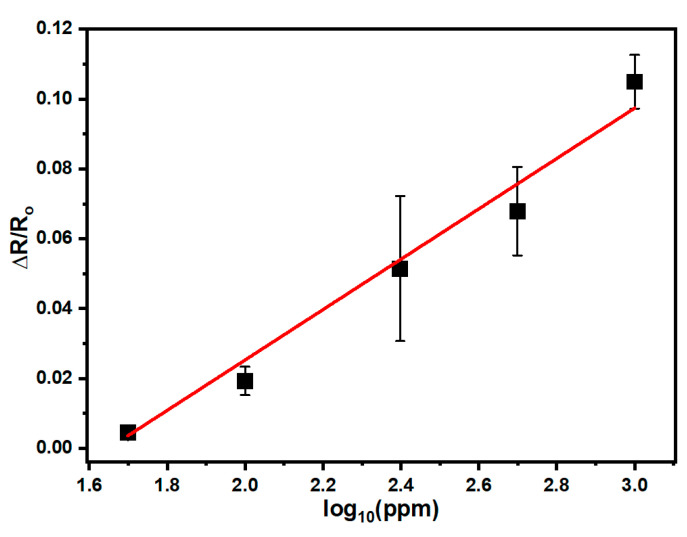
Sensitivity calibration curve for butanol at different concentrations for paper-based graphene–PEDOT:PSS/WS_2_ nanotube chemiresistor.

The WS_2_ nanotube-modified paper-based chemiresistor showed a better response to butanol gas, even at low concentrations, indicating the greater sensitivity of the resistor for butanol gas sensing. Figure S6 (Supplementary Materials) shows a comparison of the responses for methanol, toluene, and butanol at 50 ppm. The response to butanol gas was more regular and well-defined than the responses to methanol and toluene gases. The sensitivity of the paper-based graphene–PEDOT:PSS/WS_2_ nanotube chemiresistor was analyzed. Figure S7 (Supplementary Materials) shows the nonlinear pattern of methanol and toluene gas responses, and Figure 6 shows the near-linear pattern of the butanol gas response. Hence, the chemiresistor shows a well-defined response when exposed to butanol and has good reproducibility and fast recovery time; therefore, the fabricated paper-based graphene–PEDOT:PSS/WS_2_ nanotube chemiresistor is suitable for butanol gas sensing. We have compared the as-fabricated chemiresistor with other research work in different environments, as presented in Table 1.

Thus, the butanol response was near-linear, and it had sensitivity to the paper-based graphene–PEDOT:PSS/WS_2_ nanotube chemiresistor. The limit of detection (LOD) was calculated for the chemiresistor using Equation (4) of the calibration curve (*R*^2^ = 0.98), as shown in Figure 6. The LOD of the sensor was defined as three times the standard deviation of a blank signal response [34]. The LOD of the chemiresistor for butanol gas was calculated to be 44.92 ppm.

(5) $$\Delta R/R_{0}=0.0721\times LOG_{10}[\mathrm{VOCs}\;\mathrm{conc}.]-0.1189$$

with R^2^ = 0.98.

**Scheme 3:**
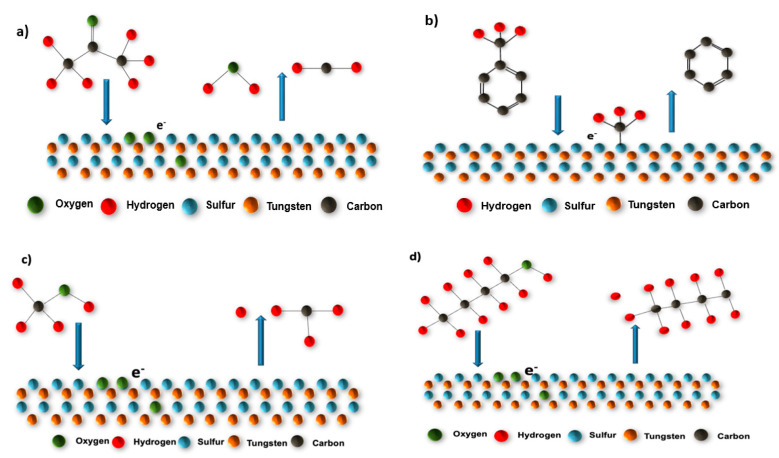
Graphical illustration of O and methyl absorption onto the surface of the WS_2_ nanotubes: (**a**) absorption of O from acetone, (**b**) absorption of methyl from toluene on the surface of WS_2_ nanotubes, (**c**) absorption of O from methanol, and (**d**) absorption of O from butanol.

The two-part sensing mechanism of this chemiresistor is proposed and illustrated in Scheme 3. The VOCs studied in this experiment were divided into two categories, namely, O-based (i.e., acetone, methanol, and butanol) and non-O-based VOCs (i.e., toluene). Besides, WS_2_ is a P-type semiconducting material that contains major carriers as holes. Under baseline conditions, the O in the atmosphere is absorbed on the surface of the WS_2_. Since sulfur in WS_2_ is bonded with weak van der Waals force, the sulfur tends to be easily replaced with other strong electron donors. Normal air contains 21% O. When the surface of the sensor is exposed to the VOCs, the O in the compounds which are donor-type species tend to donate electrons to the WS_2_ nanotubes. The electrons from the O donated by the VOCs to the WS_2_ reduce the concentration of the majority carriers and increase the concentration of the minority carriers, thereby decreasing the conductivity of the WS_2_ nanotubes. This phenomenon was also reported by Ko et al. [35]. O-based molecules, such as acetone, methanol, and butanol, donate electrons to the WS_2_ nanotubes by physical adsorption on the surface of the nanotube, resulting in decreased conductivity. In the case of non-O compounds, such as toluene, the mechanism is physisorption of the methyl group that forms an electron cloud on the surface of the WS_2_ nanotubes, thereby increasing the minority carriers and reducing the conductivity. This is consistent with findings reported by Chen et al. [36]. When the sensor was exposed to butanol gas, the resistor’s response was fast and did not reach the level of saturation, even at low concentrations, showing that the sensor had an increased ability to absorb O molecules. The vapor pressure of the analytes influences the gas sensing. The partition coefficient of the VOCs is inversely proportional to the vapor pressure, which causes the large response for the low vapor pressure gases, as reported by Hierlemann et al. [37]. In our work, we studied acetone, methanol, toluene, and butanol gases. Among these gases, butanol is a low-vapor analyte, so it produces a high response compared to other gas responses. As long as the sensor is exposed to the VOCs, the O donates more and more electrons to the WS_2_, which causes a variation in resistance.

## 4. Conclusions

In this work, we reported on the successful fabrication of WS_2_ nanotubes and flexible paper-based graphene–PEDOT:PSS/WS_2_ nanotube chemiresistors for VOC sensing at room temperature. The synthesized WS_2_ nanotubes are multiwall nanotubes of 206.9–318 nm in diameter, which was confirmed by FE-SEM and TEM images. The as-fabricated chemiresistor was tested using acetone, methanol, toluene, and butanol gases in different concentrations (50, 100, 250, 500, and 1000 ppm). The chemiresistor’s responses to methanol, toluene, and butanol were considerably higher than for acetone. The paper-based graphene–PEDOT:PSS/WS_2_ nanotube chemiresistor presented a 136% higher response to butanol than to methanol. The response to butanol gas was 42% higher than for toluene gas. Even at a concentration of 50 ppm, the sensor showed considerable variation in the response to butanol gas. The limit of detection for butanol gas was calculated to be 44.92 ppm. The results show that the paper-based graphene–PEDOT:PSS/WS_2_ nanotube chemiresistor can sensitively detect the presence of butanol gas, and has fast recovery time and good reproducibility towards butanol gas compared to other VOC gases at room temperature. The authors believe that by modifying the surface of the nanotubes with polymers, we could significantly improve the sensing performance at room temperature, and this will be the direction of our future work.

## Figures and Tables

**Table 1:** Comparison of sensing parameters with other literature and present work.

Composites	Sensing Measurement Temperature (℃)	Sensitivity to Butanol	Recovery Time (s)	Response Time (s)	Lower Detection Concentration	LOD	Ref.
ZnO@TiO_2_ NR	200	yes	26	17	0.5 ppm	133 ppb	[30]
ZrO_2_/ZnO	245	yes	40	20	20 ppm	-	[31]
Sb/ZnFe_2_O_4_	250	yes	70	7	2 ppm	30 ppb	[32]
Fe_2_O_3_/MoO_3_ nanobelts	233.5	yes	102	4	10 ppm	-	[33]
graphene–PEDOT:PSS/WS_2_ NT	RT	yes	20	205	50	44.92 ppm	This work

## Supplementary Materials

The following are available online at https://www.mdpi.com/article/10.3390/s21186121/s1, Figure S1: Completely fabricated paper-based graphene–PEDOT:PSS/WS2 nanotube chemiresistor, Figure S2: The magnified TEM image of as-synthesized WS2 multiwall nanotube. The arrows indicate the boundary of the WS2 nanotubes, Figure S3: (a) XRD pattern for synthesized WS2 nanotubes and (b) Quantitative analysis of as-synthesized WS2 con-firms the presence of W, O, and S, Figure S4: (a) Cyclic response for Acetone gas for different concentrations (b) Cyclic response for Methanol gas for different concentrations (c) Cyclic response for Butanol gas for different concentrations (d) Cyclic response for Toluene gas for different concentrations, Figure S5: Histogram of comparison between Methanol, Toluene and Butanol gas at five different concentrations, Figure S6: Low concentration response comparison between Butanol, Methanol and Toluene VOC gas, Figure S7: Selectivity calibration curve between Methanol, and Toluene VOC gas, Table S1: Response time for the all the different concentration.
