# Supplementary material for: Tungsten Disulfide Nanotube-Modified Conductive Paper-Based Chemiresistive Sensor for the Application in Volatile Organic Compounds’ Detection
Source: Sensors (Basel). 2021 Sep 12;21(18):6121. doi: 10.3390/s21186121 (PMC8472791; doi:10.3390/s21186121)
Supplement: Supplementary file 1 [file sensors-21-06121-s001.zip › sensors-1309163-supplementary.pdf]

sensors

MDPI

Supplementary Material

Tungsten Disulfide Nanotube-Modified Conductive Paper-Based Chemiresistive Sensor for the Application in Volatile Organic Compounds' Detection

Song-Jeng Huang [1], Philip Nathaniel Immanuel [1], Yi-Kuang Yen [2,*], Ching-Lung Yen [2], Chi-En Tseng [2], Guan-Ting Lin [2], Che-Kuan Lin [2] and Zhong-Xuan Huang [2]

[1] Department of Mechanical Engineering, National Taiwan University of Science and Technology, Taipei 106, Taiwan; sgjghuang@mail.ntust.edu.tw (S.-J.H.); D10603816@mail.ntust.edu.tw (P.N.I.)
[2] Department of Mechanical Engineering, National Taipei University of Technology, Taipei 106, Taiwan; t106300310@ntut.org.tw (C.-L.Y.); t106300308@ntut.org.tw (C.-E.T.); t106300324@ntut.org.tw (G.-T.L.); t106300317@ntut.org.tw (C.-K.L.); t106300304@ntut.org.tw (Z.-X.H.)
* Correspondence: ykyen@ntut.edu.tw

Citation: Huang, S.-J.; Immanuel, P.N.; Yen, Y.-K.; Yen, C.-L.; Tseng, C.-E.; Lin, G.-T.; Lin, C.-K.; Huang, Z.-X. Tungsten Disulfide Nanotube-Modified Conductive Paper-Based Chemiresistive Sensor for the Application in Volatile Organic Compounds' Detection. *Sensors* **2021**, *21*, 6121. https://doi.org/10.3390/s21186121

Academic Editors: Annanouch Fatima Ezahra and Zouhair Haddi

Received: 6 July 2021
Accepted: 9 September 2021
Published: 12 September 2021

Publisher's Note: MDPI stays neutral with regard to jurisdictional claims in published maps and institutional affiliations.

Copyright: © 2021 by the authors. Licensee MDPI, Basel, Switzerland. This article is an open access article distributed under the terms and conditions of the Creative Commons Attribution (CC BY) license (http://creativecommons.org/licenses/by/4.0/).

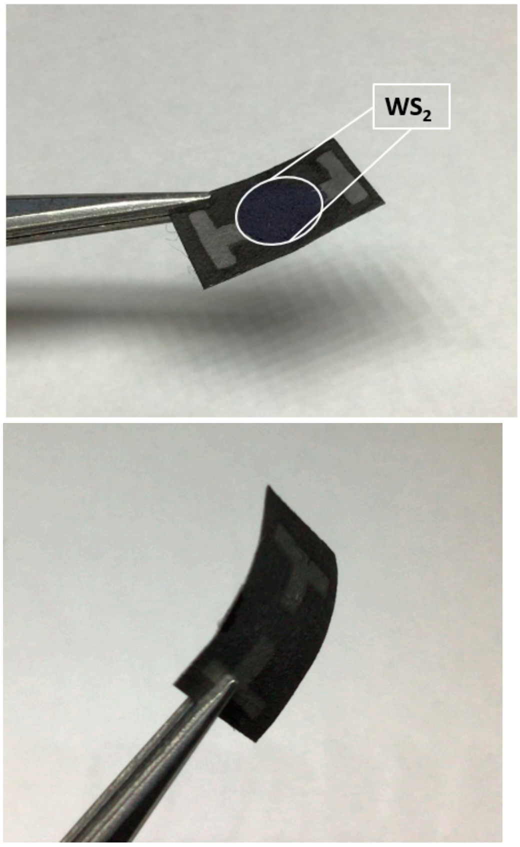

Figure S1. Completely fabricated paper-based graphene–PEDOT:PSS/WS_2 nanotube chemiresistor.

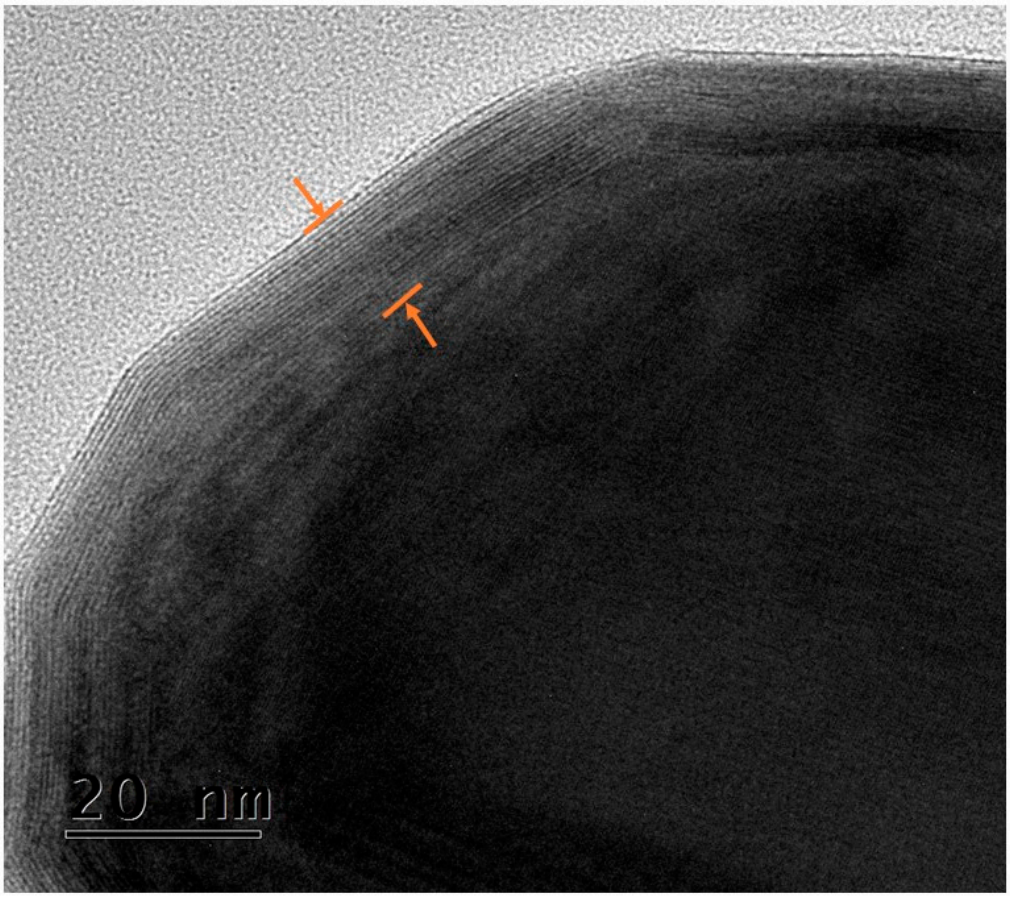

Figure S2. The magnified TEM image of as-synthesized WS_2 multiwall nanotube. The arrows indicate the boundary of the WS_2 nanotubes.

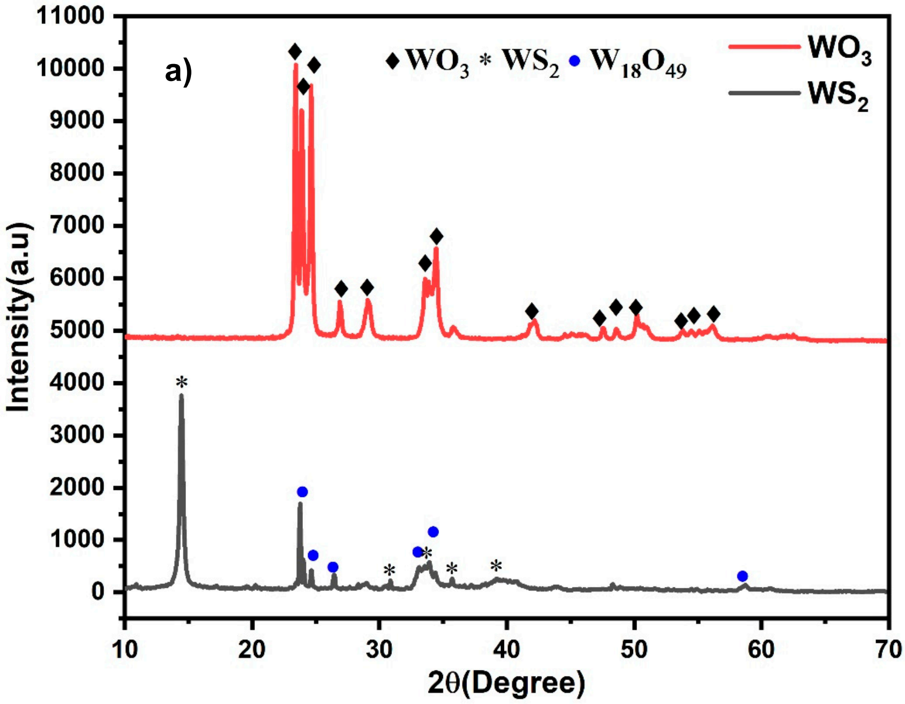

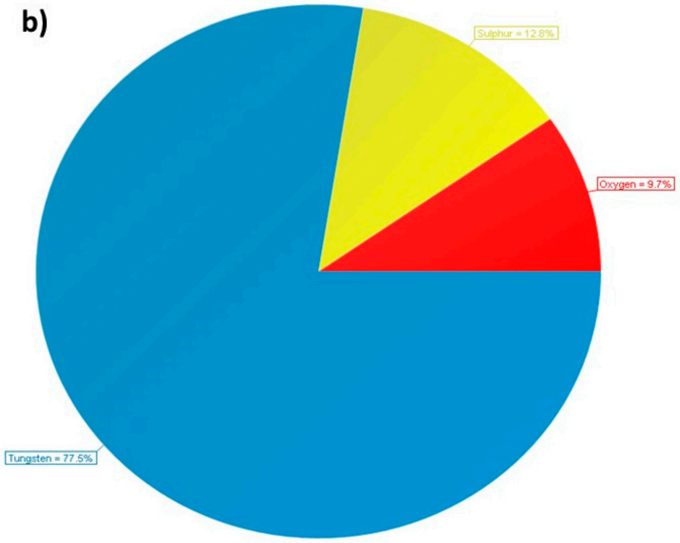

Figure S3. (**a**) XRD pattern for synthesized WS_2 nanotubes and (**b**) Quantitative analysis of as-synthesized WS_2 confirms the presence of W, O, and S.

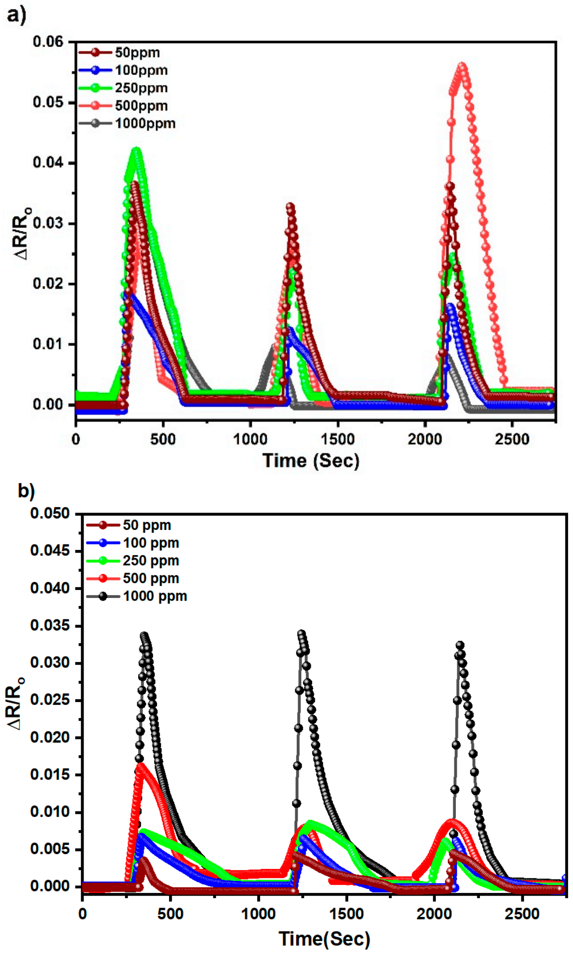

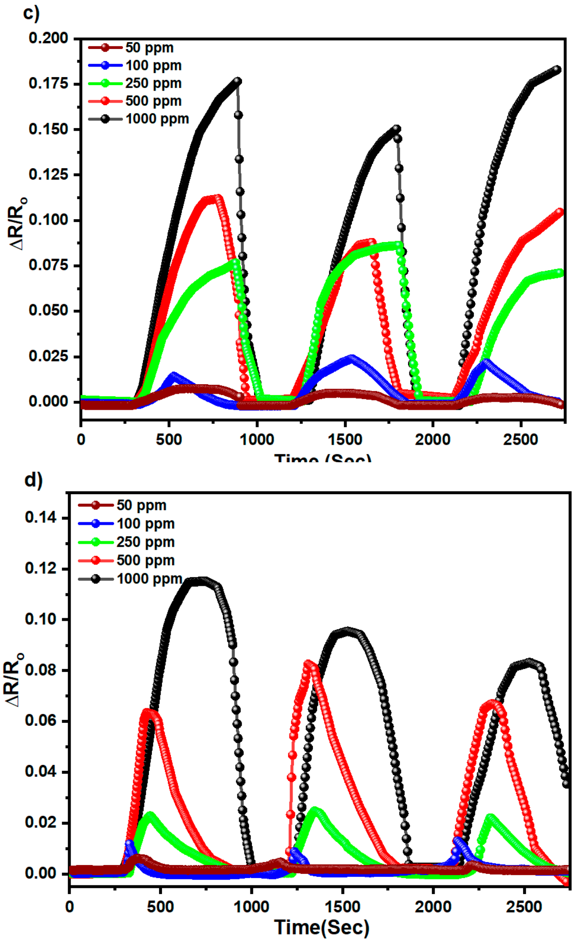

Figure S4. (**a**) Cyclic response for Acetone gas for different concentrations (**b**) Cyclic response for Methanol gas for different concentrations (**c**) Cyclic response for Butanol gas for different concentrations (**d**) Cyclic response for Toluene gas for different concentrations.

Table S1. Response time for the all the different concentration.

	1000 ppm	500 ppm	250 ppm	100 ppm	50 ppm
Acetone	72 s	72 s	103 s	82 s	98 s
Methanol	91 s	73 s	67 s	41 s	28.6 s
Toluene	201 s	194 s	160 s	137 s	134 s
Butanol	369 s	291 s	352 s	341 s	442 s

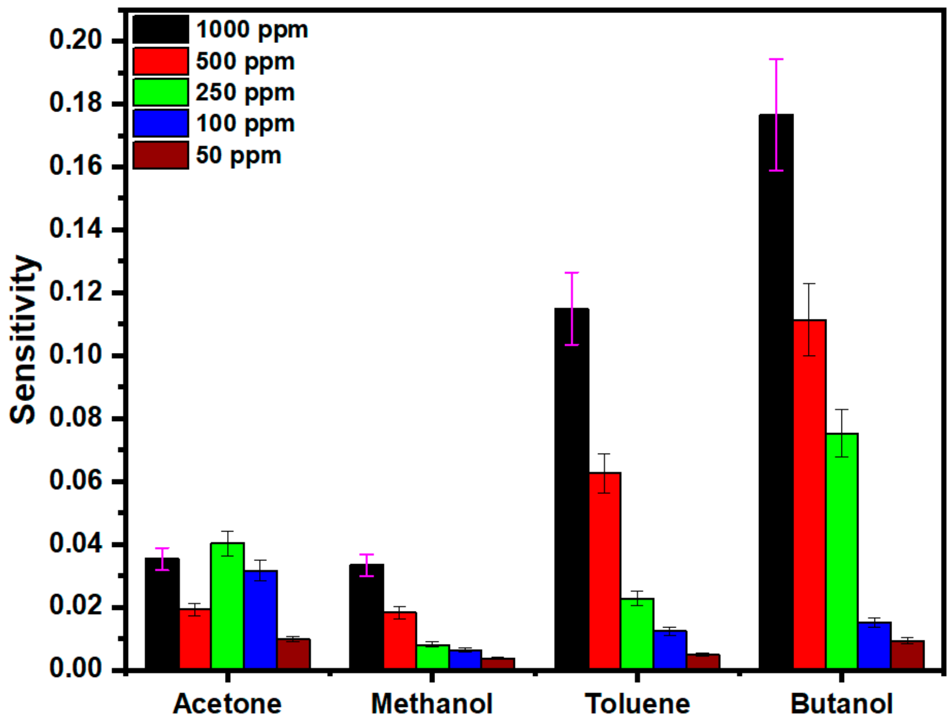

Figure S5. Histogram of comparison between Methanol, Toluene and Butanol gas at five different concentrations.

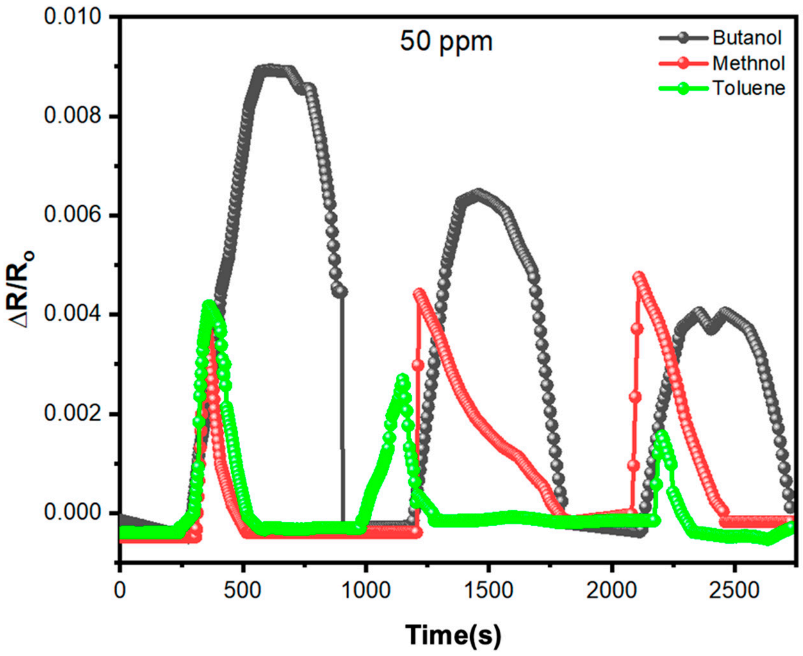

Figure S6. Low concentration response comparison between Butanol, Methanol and Toluene VOC gas.

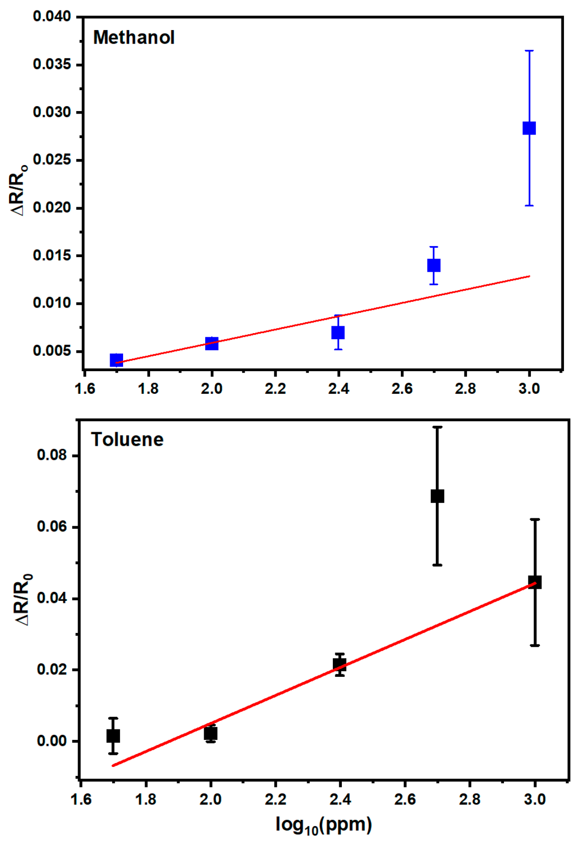

Figure S7. Selectivity calibration curve between Methanol, and Toluene VOC gas.
